# Female genital schistosomiasis, human papilloma virus infection, and cervical cancer in rural Madagascar: a cross sectional study

**DOI:** 10.1186/s40249-023-01139-3

**Published:** 2023-09-25

**Authors:** Jean-Marc Kutz, Pia Rausche, Tahinamandranto Rasamoelina, Sonya Ratefiarisoa, Ravo Razafindrakoto, Philipp Klein, Anna Jaeger, Rivo Solotiana Rakotomalala, Zoly Rakotomalala, Bodo Sahondra Randrianasolo, Sandrine McKay-Chopin, Jürgen May, Rapahel Rakotozandrindrainy, Dewi Ismajani Puradiredja, Elisa Sicuri, Monika Hampl, Eva Lorenz, Tarik Gheit, Rivo Andry Rakotoarivelo, Daniela Fusco

**Affiliations:** 1 Department of Infectious Disease Epidemiology, Bernhard-Nocht Institute for Tropical Medicine (BNITM), Hamburg, Germany; 2 German Center for Infection Research (DZIF), Hamburg-Borstel-Lübeck-Riems, Germany; 3 Centre Infectiologie Charles Mérieux (CICM), Antananarivo, Madagascar; 4 Centre Hospitalier Universitaire (CHU) Androva, Mahajanga, Madagascar; 5 Association K’OLO VANONA, Antananarivo, Madagascar; 6 International Agency for Research on Cancer (IARC), Lyon, France; 7 University Medical Center Hamburg-Eppendorf (UKE), Hamburg, Germany; 8 University of Antananarivo, Antananarivo, Madagascar; 9 Barcelona Institute for Global Health (IS Global), Barcelona, Spain; 10 Köln-Hohenlind Hospital, Cologne, Germany; 11 University of Fianarantsoa, Fianarantsoa, Madagascar

**Keywords:** Women’s health, Female genital schistosomiasis, Human papilloma virus, Cervical cancer, Public health, Madagascar

## Abstract

**Background:**

Women’s health in resource-limited settings can benefit from the integrated management of high-burden diseases, such as female genital schistosomiasis (FGS) and human papilloma virus (HPV)-related cervical cancer. In schistosomiasis-endemic countries such as Madagascar, data on FGS and HPV prevalence are lacking as well as preventive measures for both conditions. This study aims to estimate the prevalence of FGS and HPV in rural Madagascar, and to examine associated risk factors to identify opportunities for improving women’s health.

**Methods:**

After initial community outreach activities, interested women aged 18–49 years were recruited consecutively in 2021 at three primary health care centers in the district of Marovoay. FGS was detected by colposcopy. Colposcopy images were double-blind reviewed by two independent specialists. A Luminex bead-based assay was performed on cervical vaginal lavage specimens for HPV typing. Crude (CPR) and adjusted prevalence ratios (APR) of associations between selected factors and FGS and HPV positivity were estimated using univariable and multivariable binary Poisson regression with 95% confidence intervals (*CI*s).

**Results:**

Among 500 women enrolled, 302 had complete information on FGS and HPV diagnosis, and were thus eligible for analysis. Within the sample, 189 (62.6%, 95% *CI*: 56.9–68.1) cases of FGS were detected. A total of 129 women (42.7%, 95% *CI*: 37.1–48.5) tested positive for HPV. In total, 80 women (26.5%, 95% *CI*: 21.6–31.8]) tested positive for both conditions. No association was observed between FGS and HPV positivity, while previous pregnancy (APR = 0.65, 95% *CI*: 0.43–0.78) and older age (APR = 0.59, 95% *CI*: 0.42–0.81) are showing a negative association with HPV infection compared to no previous pregnancy and younger age groups.

**Conclusions:**

The results of the study show that FGS and HPV are highly prevalent in rural Madagascar. The concurrent prevalence of these two conditions requires urgent adaptations of public health strategies to improve women’s health, such as integrated services at primary level of care.

**Graphical Abstract:**

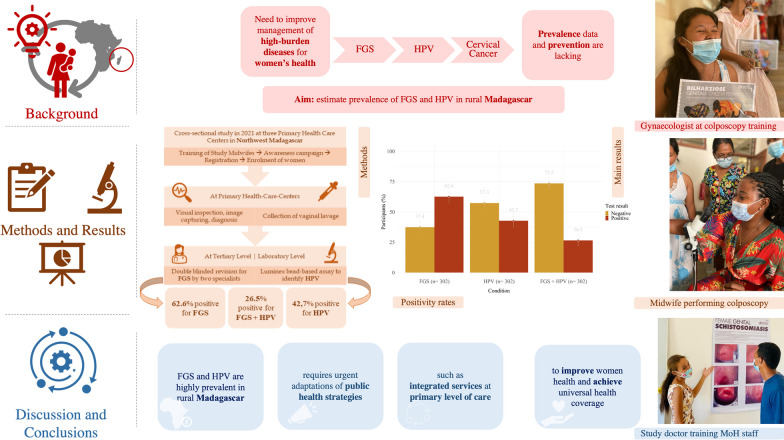

**Supplementary Information:**

Theonline version contains supplementary material available at 10.1186/s40249-023-01139-3.

## Background

Health is a human right and, as such, health care should be accessible to all, regardless of gender, or geographical location. In the African Region, women are more likely to die from communicable diseases and other conditions than women in other regions [1, 2]. Additionally, it has been reported that one in four deaths among adult women are attributable to non-communicable diseases (NCDs), the attributable burden of which is more strongly associated with infectious agents in sub-Saharan Africa (SSA) than in other regions of the world [3]. The Sustainable Development Goals (SDGs) consider women's health as one of the main health-related themes, with a strong emphasis on maternal and sexual and reproductive health [4]. Shifts in population dynamics towards a more ageing population create new challenges and greater complexities in the global burden of diseases, including an increase in NCDs [5].

Female genital schistosomiasis (FGS) [6] and cervical cancer (CC) [7] are two examples of chronic conditions, caused by exposure to risk factors at earlier stages of life, and affecting women in SSA disproportionately more than in other regions of the world [8, 9].

FGS is a common consequence of schistosomiasis, a parasitic disease caused, in its urogenital form, by the trematode *Schistosoma haematobium* [10]. The deposition of schistosome’s eggs into urogenital tissue perpetuates inflammatory and proinflammatory responses leading to the formation of granulomas typically in the bladder wall [11, 12]. Chronic infections with *S. haematobium* can also culminate in FGS, leading to ectopic pregnancy, infertility, cervical lesions, and not last, social stigma [6]. The worldwide burden of FGS is mostly unknown, though it is described that between 15 and 70% of human schistosomiasis cases can lead, if untreated, to FGS [6]. Human schistosomiasis is highly prevalent in SSA where more than 90% of those requiring treatment live [13].

Schistosomiasis control strategies in highly endemic areas are mostly based on preventive chemotherapy through mass drug administration (MDA) with 40 mg/kg praziquantel (PZQ) for school aged children [14]. In contrast, the recent World Health Organization (WHO) guidelines suggest treatment for all above 2 years of age in highly endemic areas [15]. In addition to organised MDAs, treatment for schistosomiasis is formally offered in endemic countries, such as in Madagascar [16], through health counselling but often based on an out-of-pocket payment principle leaving de facto a relevant part of the infected population untreated and susceptible to developing chronic forms of the disease such as FGS. The treatment with a single dose of PZQ has been proven to have a limited effect on the resolution of the lesions produced by the egg deposition and host-inflammatory response [12]. Moreover, frequent re-exposure to the pathogen and long-standing infections may play a role in the lesion’s aggravation [11]. New evidence suggests that FGS lesions also represent a risk factor for sexually transmitted viral infections such as HIV [17, 18]. FGS remains often undetected since the diagnosis mostly relies on visual inspection of characteristic lesions (i.e., yellow sandy patches, abnormal vessels and rubbery papules) on the cervix and vaginal wall that can be visualised through colposcopy [19]. At this stage of the disease, in fact, schistosome eggs are mostly not released into urine [20] making microscopy unsuitable for the detection of FGS [21, 22]. Colposcopy is an advanced gynaecological investigation that requires an experienced investigator and is scarcely available at primary level of care and in general in low- and middle-income countries (LMICs) where its use is being debated for decades because of standardization challenges and performances variability [23, 24].

Colposcopy is also a key clinical diagnostic tool for the prevention and management of CC [25]. CC is the second most common cancer among women with approximately 90% of the deaths occurring in LMICs in 2020 [26, 27]. Nearly all cases of CC can be attributed to human papilloma virus (HPV) infection, even acquired at young age, making CC one of the most preventable cancers [27, 28] overall. HPV is the most common sexually transmitted virus worldwide [27] with more than 100 different human types organized into five major genera: alpha, beta, gamma, mu, and nu [29]. HPV types are commonly divided into high (carcinogenic) or low-risk (non-carcinogenic) types [30]. Hence, twelve alpha mucosal HPV types (HPV 16, 18, 31, 33, 35, 39, 45, 51, 52, 56, 58, and 59), referred to as high-risk HPV types (HR-HPV), were classified as carcinogenic to humans. Eight other alpha HPV types (HPV 26, 53, 66, 67, 68, 70, 73, and 82) were classified as probably or possibly carcinogenic [31]. CC is the most frequent type of cancer associated with HPV infection and is almost always associated with HR-HPV types [32]. CC can be controlled through primary (HPV vaccination), secondary (cervical screening and treatment of precancerous lesions) and tertiary (early diagnosis and treatment of cancer) prevention which should be combined to reduce morbidity and mortality [33]. In LMICs, primary prevention is even more critical than in other regions of the world because of the scarcity of services and infrastructures capable to properly manage advanced forms of the disease [34, 35]. However, the implementation of preventive measures and population-based screening programmes in LMICs in general, and in SSA in particular, has been shown to be challenging due to financial, logistic, and socio-cultural factors [36]. In November 2020, the WHO launched a global initiative to eliminate CC as a public health problem through the implementation of the 90/70/90 triple intervention strategy. The strategy aims to vaccinate at least 90% of girls against HPV by the age of 15 years, to screen 70% of women using a high-performance test by the age of 35 years and again by the age of 45, and to treat at least 90% of identified precancerous lesions and invasive cancers [37].

At the current state of the art, few studies have explored the associations of FGS, HPV infection and CC [38]. Though, given the physio-pathological progression of the conditions, it is legitimate to assume associations among them.

Among SSA-countries, Madagascar is one of the countries with the highest prevalence of schistosomiasis [39–41]. CC is the most frequent case of cancer in the country and the most common cause of cancer deaths [42]. FGS, HPV and CC are suspected to be highly prevalent in the country though little is known about the medical needs associated with these diseases. Consequently, this hampers the adaptation of guidelines and health services for prevention, control and management [43].

This study is based on data collected in the Boeny region of Madagascar, known to be highly endemic for *S. haematobium* [39]. To our knowledge, this is the first study describing the prevalence of FGS, HPV, and their co-presence in the region. Our findings aim to provide prevalence estimates and to describe associations between risk factors and FGS and HPV infection to inform control strategies for FGS and CC, align the country with the global health agenda, and promote overall improvement of women’s health.

## Methods

### Study desgin, area and population

This cross-sectional study was conducted at three Primary Health Care Centres (PHCCs) in the district of Marovoay in the Boeny region of Madagascar: PHCC of Antanambao-Andranolava (15°58′00″S, 46°41′00″E), PHCC of Ankazomborona (16°06′50″S, 46°45′24″E) and PHCC of Marovoay-Ville (16°06′40″S, 46°38′38″E). Available data from health districts do not allow the classic categorisation based on the degree of urbanisation [44], although Antanambao-Andranolava can be described best as rural, Ankazomborona as rural and Marovoay as peri-urban [45] according to local characteristics.

The study area has been selected due to their estimated prevalence of more than 50% of *S. haematobium* in the adult population and a population at risk of 25% to acquire FGS [39]. An estimated 543,200 people live in the region [45]. The three PHCCs have a catchment area of around 60,000 people, of whom 23,000 live in the ten communities of Ankazomborona, 3000 in the eight communities of Antanambao-Andranolava and 34,000 in the town of Marovoay [45].

### Recruitment and eligibility criteria

Following an awareness campaign from September 2020 to February 2021, enrolment lists were made available at the three PHCCs, where women had four months to express their interest in participating in the study. Women in the study area were contacted and invited to the PHCCs for a gynaecological consultation on a first-come, first-served basis, and the first 500 registered women were enrolled in the study. In order to assess overall adherence to the service, all women who expressed an interest in participating were registered. Eligibility criteria were: (i) aged between 18 and 49 years; (ii) fluent in French or Malagasy; and (iii) voluntary written informed consent. Exclusion criteria for the study was pregnancy.

### Data collection and data management

Participants were recruited between December 2020 and February 2021, with examinations and data collection taking place between March and August 2021. Prior to gynaecological examinations and sample collection, six study midwives were trained to perform colposcopy for diagnosing FGS. At recruitment, each participant was assigned a unique identifier to ensure anonymous identification and attribution to each image captured. To each sample, a unique corresponding sample identifier was assigned. Conformity and quality control were performed at laboratory level prior to storage. Background characteristics of the participant, such as socio-demographic information, clinical history, and personal habits were captured in case report form (CRF).

All CRFs were checked for missing entries manually by study midwives and researchers following standard operating procedures. Double data entry was used to feed the REDCap^®^ database [46, 47]. Quality control of data processing and validation was undertaken in regular intervals during and at the end of data entry.

### Gynecological investigation and sample collection

After insertion of a disposable plastic speculum, the gynecological examination was performed in three steps: (i) cervicovaginal lavage (CVL); (ii) visual inspection; and (iii) image capture. Inspection of the mucosal surfaces was performed with the colposcope based on an adapted protocol from Singer and Monaghan [48]. At each examination, one colposcopic image (COLIM) was captured. Consequently, a sample of 10 ml of CVL was collected in tubes containing liquid-based cytology medium (ThinPrep®) according to the manufacturer's instructions (Hologic, Marlborough, Massachusetts, USA) [49]. CVLs were collected by swabbing the cervix with a brush, and then placed with liquid into ThinPrep^®^ collection tubes. Samples were stored according to standard conditions [49] before shipment to Europe for analysis.

All images were reviewed within twelve hours by an experienced gynaecologist. All women suspected of any gynaecological condition different from FGS were invited to the university hospital of Mahajanga for further investigations. Standard treatment for FGS (single dose of PZQ 40 mg/kg) was offered free of charge for all diagnosed pathologies.

### FGS definition and diagnosis

At the PHCCs, women were considered positive for FGS if the study midwives identified one of the four cervicovaginal manifestations described in the WHO FGS atlas [50]**:** (i) abnormal blood vessels; (ii) rubbery papules; (iii) homogeneous yellow sandy patches; and/or (iv) grainy sandy patches on colposcopy. The final diagnosis of FGS was determined by a post-recruitment evaluation of the COLIM revised through a double-blinded assessment of two FGS expert gynecologists (ZR and BR). The presence of one or more FGS signs as described previously was required to define a COLIM as positive. FGS negativity was defined as absence of those signs. Three categories were defined for classification of images: positive, negative, or indeterminate for FGS. Images that received a concordant rating were classified as either negative or positive and were included in analyses. Discordant ratings were classified as indeterminate and excluded from analyses. The presence of one or more FGS sign as described previously was required to define a COLIM as positive. FGS negativity was defined as no presence of those signs.

### HPV analysis

CVL were analyzed at the International Agency for Research on Cancer (IARC) in Lyon, France. There, a standardized methodology aimed at identifying all HPV strains has been established and used for worldwide HPV surveillance [51–53]. Samples were analysed as described in Schmitt et al. [51] and briefly: First, the presence of beta-globin was checked on CVL samples as a quality control. One aliquot of 1 ml *ThinPrep* media was centrifuged at 3300 × *g* for 10 min to pellet the cervical exfoliated cells. After removing the supernatant, DNA extraction was performed using the Qiagen BioRobot EZ1 with the EZ1 DNA tissue kit according to the manufacturer's instructions (Qiagen, Hilden, Germany). DNA was eluted in 100 μl of elution buffer [51, 54].

The presence of HPV DNA was detected using a type-specific E7 PCR bead-based multiplex genotyping assay (E7-MPG, IARC, Lyon, France). The E7-MPG assay utilizes HPV type-specific primer pairs targeting the E7 region of 19 probable/possible high-risk (pHR) or high-risk (HR) HPV types (HPV-16, -18, -26, -31, -33, -35, -39, -45, -51, -52, -53, -56, -58, -59, -66, -68a and b, -70, -73, and -82) and two low-risk HPV types (HPV-6 and -11), plus primers for the amplification of a b-globin sequence [51, 53–55]. 10 μl of DNA extracted from CVL have been used to perform the PCR.

Following PCR amplification, 10 μl of each reaction mixture were analyzed by MPG using the Luminex technology as described previously (Luminex Corporation, Austin, Texas, USA) [56]. Briefly, the reporter fluorescence was quantified using Luminex reader 200 (Luminex Corporation), and cutoffs were computed by adding 5 to 1.1 multiplied by the median background value expressed as median fluorescence intensity [51, 52, 54].

Overall and type-specific HPV prevalence was estimated as the proportion of patients who tested HPV DNA positive for a given HPV DNA type.

### Statistical analysis

All analyses were conducted using R^®^ v.4.2.2 [57]**.** Continuous variables were described using means (with standard deviation) and medians (with interquartile ranges). Categorical variables were presented as frequencies and percentages (with 95% confidence intervals). Only complete cases were considered for analyses.

Positivity rates of FGS, HPV and their co-presence were reported as proportions with exact 95% confidence intervals (*CI*s). Presence and combinations of possible FGS symptoms were described among FGS positives and negatives.

Three distinct risk factor analyses were performed based on the diagnostic results for FGS, HPV, and co-presence of the two conditions. Test result distribution among individuals with various risk factors (study site, sex, age, education level, ever been treated with PZQ, working as a farmer and being the main contributor to the family income) was described. Univariable and multivariable binary Poisson regression (with robust standard errors for confidence interval calculation) were performed to derive crude and adjusted prevalence ratios with 95% *CI*s.

### Ethical consideration

Ethical approval was obtained from the National Ethics Committee of Madagascar (ref. no. N°052-MSANP/CERBM) and the Ethics Committee Hamburg State Medical Chamber, Germany (ref. no. PV7309).

All participants were informed of the aims and procedures of the study in Malagasy language. Participation in the study was voluntary, and informed consent for participation was obtained from the participant by signature or, in the case of illiteracy, by thumbprint in the presence of an independent witness. In all cases, participants had the right to refuse to participate and to withdraw informed consent at any time without giving reasons. No financial incentives were given for participation in the study. Diagnosed pathologies were treated according to national guidelines and free of charge.

## Results

### Recruitment and sample selection

**Fig. 1:**
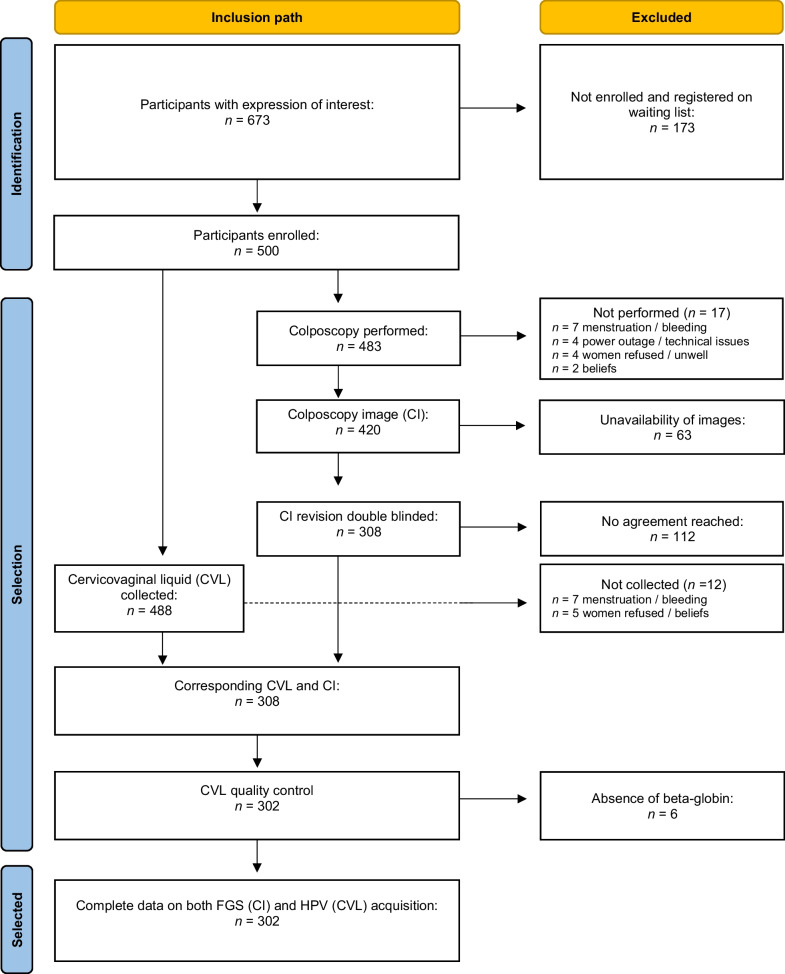
Sampling and inclusion flow [58]

A total of 673 women expressed interest in taking part in the study (Fig. 1). Among these, 500 were enrolled while the remaining 173 women were placed on a waiting list based on the protocol’s maximum recruitment number. Of those enrolled, 483 underwent colposcopy and 488 colposcopy investigation plus CVL. In 17 cases, colposcopy could not be performed because of menstruation (*n* = 7), technical problems (*n* = 4) such as power failure, because the woman was uncomfortable with the procedure and refused (*n* = 4), or because of religious or cultural beliefs (*n* = 2). CVL could not be performed in 12 cases because the woman was menstruating or otherwise bleeding (*n* = 7) or less frequently because of religious or cultural beliefs or fear of the procedure (*n* = 5). Among the images captured via colposcopy 63 images were not interpretable because of poor image quality (i.e., blurred images) or because of lack of ID correspondence. After double-blinded revision, a total of 112 COLIMs were excluded from the analysis because a consensus between FGS positive or negative classification could not be reached. After CVL quality control 302 corresponding COLIMs and CVLs were obtained with complete data on both FGS and HPV acquisition.

### Study population

A total of 302 women were considered for analysis. The median age of the participants was 31 years (IQR: 25–37) (Table 1), ranging from 18 to 49 years. In Marovoay (peri-urban community MVY) 63.0% (*n* = 89) of participants had completed secondary school or higher, while in the rural community of Antanambao-Andranolava (ATA), 75.0% (*n* = 83) of participants had completed primary school.

**Table 1:** Socio-demographic background of study participants

PHCC	Antanambao Andranolava (ATA-rural)	Ankazomborona (AKZ-rural)	Marovoay (MVY-peri-urban)
	*n* = 111 (100%)	*n* = 50 (100%)	*n* = 141 (100%)
Age in years^a^	31.0 (37.0–22.0)	31.5 (39.5–26.3)	31 (39.0–25.0)
Education			
None	18 (16.2)	6 (12.0)	10 (7.1)
Primary education	83 (74.8)	20 (40.0)	42 (29.8)
Secondary education or higher	10 (9.0)	24 (48.0)	89 (63.1)
Main contributor family income			
Me	30 (27.0)	4 (8.0)	16 (11.3)
Husband	77 (69.4)	38 (76.0)	113 (80.1)
Other^b^	4 (3.6)	8 (16.0)	12 (8.5)
Employment status			
Unemployed	1 (0.9)	2 (4.0)	17 (12.1)
Student	0 (0.0)	2 (4.0)	4 (2.8)
Employee	5 (4.5)	10 (20.0)	24 (17.0)
Self employed	105 (94.6)	36 (72.0)	96 (68.1)
Profession^c^			
Farmer	95 (85.6)	30 (60.0)	48 (34.0)
Non-farmer	16 (13.5)	20 (32.0)	93 (51.1)

^a^Median (IQR)

^b^Other include Uncle, Aunt, Mother, Father

^c^Non-farmers includes students and unemployed

In total 78.0% (*n* = 237) of the participants were self-employed and the most common occupation among the women was farming with 57.0% (*n* = 173) (Table 1). While 85.6% (*n* = 95) of employed participants in the rural community ATA were farmers, only 34.0% (*n* = 48) in the urban community MVY were employed in this sector. While in the rural community almost all participants were employed with 99.0% (*n* = 110) in ATA and 92.0% (*n* = 46) in Ankazomborona (rural community AKZ), in the peri-urban community 12.1% (*n* = 17) were unemployed. Across all sites 76.0% (*n* = 228) of participants reported that their husbands as the main contributors to the family income (Table 1).

While 87.2% (*n* = 123) of participants in the peri-urban community MVY reported to never have had treatment with PZQ, 54.1% (*n* = 60,) participants from the rural community ATA and 44.0% (*n* = 22) of the rural community AKZ reported to have been treated with PZQ prior to our study (Table 2). The majority of participants reported overall a previous pregnancy and 45.4% (*n* = 137) participants reported a previous miscarriage. None of the participants had been vaccinated against HPV (Table 2).

**Table 2:** Schistosomiasis clinical history and gynecological history of study participants

PHCC	Antanambao Andranolava (ATA-rural)	Ankazomborona (AKZ-rural)	Marovoay (MVY-peri-urban)
	*n* = 111 (100%)	*n* = 50 (100%)	*n* = 141 (100%)
Previous treatment with PZQ			
No	51 (45.9)	26 (52.0)	123 (87.2)
Yes	60 (54.1)	22 (44.0)	15 (10.6)
Timing of previous treatment^a^			
Last year	8 (13.3)	0 (0.0)	4 (26.7)
More than one year	52 (86.7)	6 (27.3)	8 (53.3)
Symptoms (past year)^b^			
None	29 (26.1)	29 (58.0)	42 (29.8)
1 to 2 symptoms	56 (50.4)	15 (30.0)	80 (56.8)
3 and more	26 (23.4)	6 (12.0)	19 (13.5)
Previous pregnancy			
No	11 (9.9)	3 (6.0)	12 (8.5)
Yes	100 (90.1)	47 (94.0)	129 (91.5)
Previous miscarriage^a^			
No	57 (57.0)	23 (48.9)	59 (45.7)
Yes	43 (43.0)	24 (51.1)	70 (54.3)
Number of miscarriages^a^			
1–2	40 (93.0)	22 (91.6)	60 (85.7)
3 and more	3 (6.9)	2 (8.4)	10 (14.3)
Living children^a^			
No	3 (3.0)	5 (10.6)	16 (12.4)
Yes	97 (97.0)	42 (89.4)	113 (87.6)
HPV vaccination			
No	111 (100.0)	50 (100.0)	141 (100.0)

^a^Conditional questions resulting in missing values by design

^b^Symptoms include pain, vaginal discharge, irregular bleeding, and genital itching

Participants’ smoking and alcohol consumption was also surveyed. Of the women in the peri-urban community MVY 9.4% (*n* = 17) reported to be smokers, whereas only 1.0% (*n* = 1) in the rural community AKZ, and 1.7% (*n* = 2) in the rural community AA reported to be smoking. Alcohol consumption was reported to be 34.8% in MVY, 26.0% (*n* = 13) in AKZ, and 20.7% (*n* = 23) in AA (see Table 3).

**Table 3:** Study participants’ personal risk consumption habits

PHCC	Antanambao Andranolava (ATA-rural)	Ankazomborona (AKZ-rural)	Marovoay (MVY-peri-urban)
	*n* = 111 (100%)	*n* = 50 (100%)	*n* = 141 (100%)
Alcohol consumption^a^			
No	88 (79.2)	37 (74.0)	92 (65.2)
Yes	23 (20.7)	13 (26.0)	49 (34.8)
Smoking^a^			
No	109 (98.3)	49 (99.0)	124 (90.6)
Yes	2 (1.7)	1 (1.0)	17 (9.4)

^a^Yes includes consumption on special occasions and regular consumption

### Prevalence of FGS and HPV infection

**Fig. 2:**
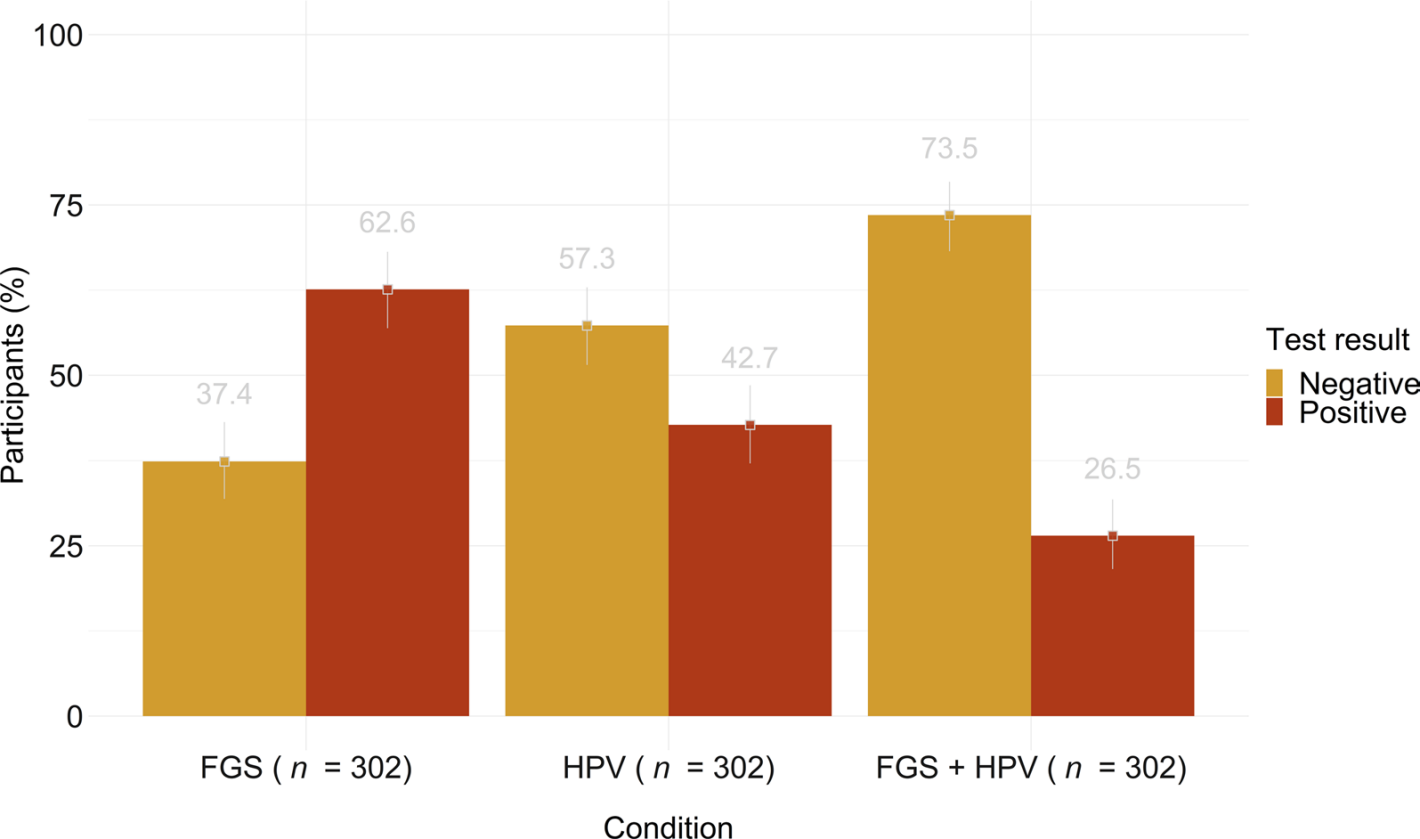
Prevalence and 95% confidence intervals for FGS; HPV and FGS + HPV from 302 women (in %)

In total, an FGS prevalence of 62.6% (*n* = 189; 95%* CI*: 56.9–68.1) was reported in the area (Fig. 2). The highest positivity rate was reported in the rural community AKZ, where 80.0% (*n* = 40) of participants were tested positive for FGS. The lowest rate was observed in the peri-urban community MVY, where 52.0% (*n* = 73) of the women were tested positive for FGS. In the remaining site in the rural community ATA, 69.0% (*n* = 76) of the women were tested FGS-positive. Three cases of suspected pre-cervical cancerous lesions were described but none of the cases was diagnosed with CC.

In total, 42.7% of women (*n* = 129; 95% *CI*: 37.1–48.5) were tested positive for HPV. The highest HPV positivity rate was reported in the rural community AKZ (46.0%, *n* = 23). The lowest rate was observed in the urban community MVY, where 40.0% (*n* = 57) of women were positive for HPV. In the rural community ATA 44.0% (*n* = 49) of the women were FGS-positive.

In addition, of all HPV infections, 18.0% (*n* = 23) reported a low to probable high risk of HPV infection and 82.0% (*n* = 106) had a high-risk infection.

In total, 26.5% (*n* = 80; 95%* CI*: 21.6–31.8) tested positive for both FGS and HPV infection. The highest rate of co-infection was found in the rural community AKZ with 36.0% (*n* = 18), and the lowest in the peri-urban community MVY (20.0%, *n* = 28). The third study site, the rural community ATA, reported 31.0% (*n* = 34) co-infection with FGS and HPV acquisition. No cases of CC were diagnosed in our study population.

### Gynaecological symptoms and FGS status

**Fig. 3:**
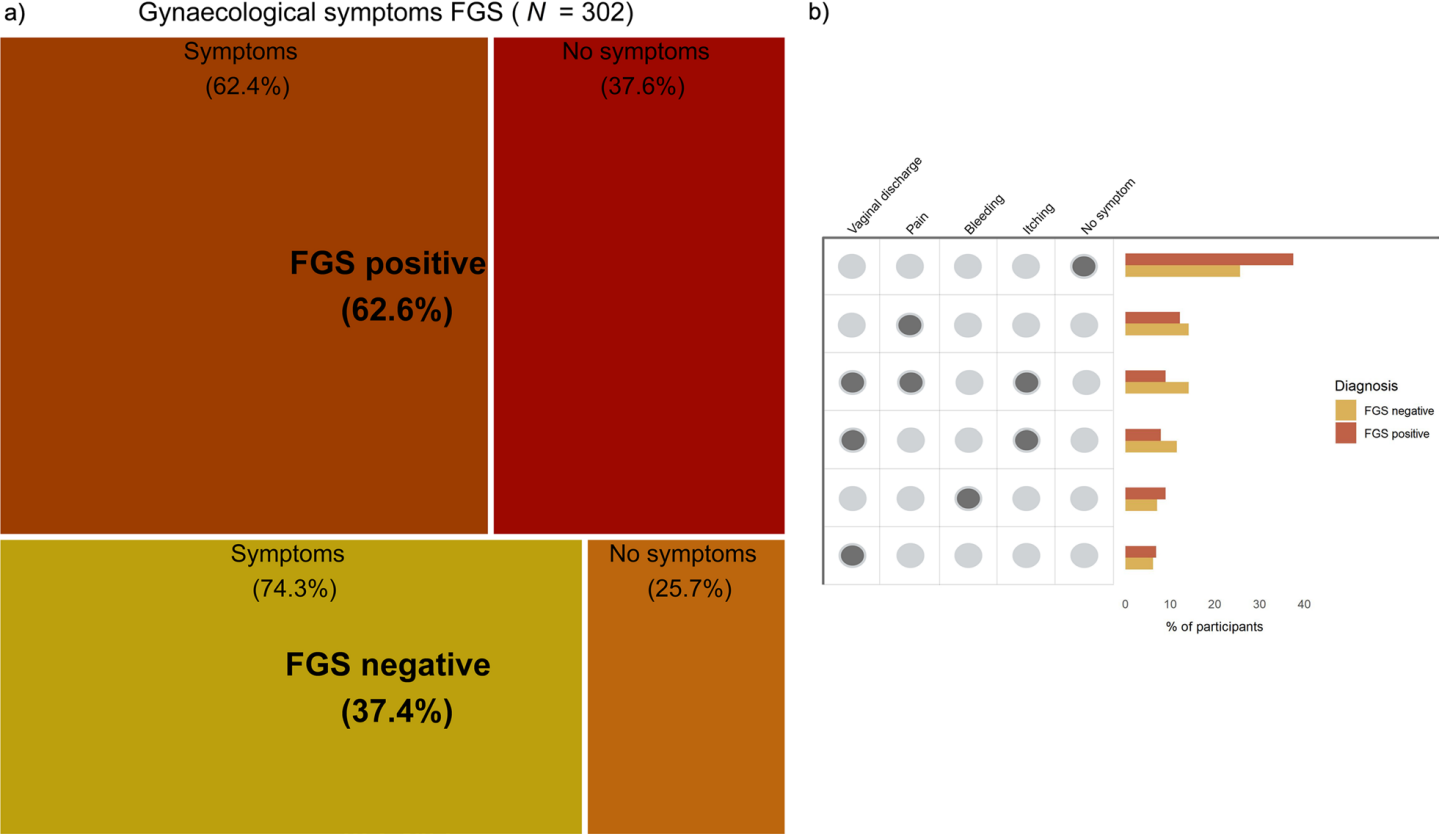
Gynaecological symptoms FGS of study participants **a** Matrix plot showing the intersection of symptoms with FGS positivity. The area of each rectangle is proportional to the number of participants in the category. Created with https://www.biorender.com. **b** Six most frequent symptom combinations among FGS positives and negatives

All participants were asked for the presence of the four most common symptoms of FGS (vaginal discharge, pain, bleeding, itching). Figure 3 depicts the six most frequent combinations as reported by FGS positive and negative women. While a total of 62.6% (*n* = 118) of women with FGS had at least one of the symptoms, 37.6% (*n* = 71) of the FGS-positive women had no symptoms. Among the FGS-negative participants, 74.3% (*n* = 84) presented symptoms, and 25.7% (*n* = 29) had no symptoms. Pain was the most frequently reported symptom as in 12.2% (*n* = 23) for FGS positive participants and 14.2% (*n* = 16) for FGS negative participants. The third most frequently reported set of symptoms was the presence of vaginal discharge, pain and itching in 9.0% (*n* = 17) of FGS positive women, and 14.2% (*n* = 16) for FGS negative women. Bleeding was reported in 7.9% (*n* = 15) for FGS negative women, and 11.5% (*n* = 13) in FGS positive women. The symptom “vaginal discharge” was reported 6.9% (*n* = 13) by FGS positive women, and 6.2% (*n* = 7) by FGS negative women.

### Prevalence ratio for FGS, HPV and both concomitant conditions

Crude and adjusted prevalence ratios (CPR and APR) were estimated for associations with selected risk factors for FGS (Additional file 1: Table S1), HPV separately (Additional file 2: Table S2) and the two conditions occurring concomitantly (Additional file 3: Table S3).

### FGS

The APR for different age groups was reported for FGS positive women and is reported in Additional file 1: Table S1.

Women in the urban community MVY trended to be less prevalent with FGS (APR = 0.76; 95% *CI*: 0.62–0.93) and more prevalent in the rural community AKZ (APR = 1.17; 95% *CI*: 0.97–1.41), when compared to the rural community ATA (Additional file 1: Table S1).

Other factors explored such as previous PZQ treatment, primary education, secondary education, and farming did not show any strong difference (Additional file 1: Table S1).

Previous pregnancy indicated an increased FGS prevalence (APR = 1.42; 95% *CI*: 0.89–2.26) compared to no previous pregnancy (*P* > 0.05). In relation to no symptoms, increasing numbers of symptoms were associated with FGS as follows: lower prevalence in women with one symptom (APR = 0.86; 95% *CI*: 0.70–1.07), two symptoms (APR = 0.99; 95% *CI*: 0.76–1.25), and with three or more symptoms (APR = 0.75; 95% *CI*: 0.56–1.00) (Additional file 1: Table S1).

### HPV

Previous pregnancy showed a 35.0% reduced prevalence of HPV infection (APR = 0.65; 95% *CI*: 0.43–0.78), when compared to women with no previous pregnancy (Additional file 2: Table S2).

Prevalence was reduced in the age group 25 to 34 (APR = 0.59; 95% *CI*: 0.42–0.81) and for the age group 35–44 (APR = 0.89; 95%* CI*: 0.64–1.24) as well as for the age group 45–49 (APR = 0.67; 95% *CI*: 0.41–1.07) when compared to the age group 18 to 24.

FGS assessed as a concomitant condition with HPV showed a slight reduction in prevalence in FGS positive women compared to FGS negative women. Women in the peri-urban community MVY showed a lower trend for HPV infection compared to the rural community ATA (Additional file 2: Table S2). Compared to no education, primary education and secondary education showed no strong trend (Additional file 2: Table S2). Farmers compared to non-farmers showed an APR of 1.14 (Additional file 2: Table S2). Alcohol consumption was also assessed with a slightly higher trend compared to no alcohol consumption, as was smoking compared to no smoking (Additional file 2: Table S2).

### FGS and HPV

There is a lower tendency for FGS-HPV co-infection among the age group 25 to 34 (APR = 0.55; 95% *CI*: 0.33–0.89), the age group 35–44 (*APR* = 0.99; 95% *CI*: 0.62–1.57), as well as for the age group 45–49 (APR = 0.62; 95%* CI*: 0.32–1.21) when compared to the age group 18 to 24.

Women in the peri-urban community MVY had less FGS-HPV co-infection and a higher APR in the rural community AKZ, when compared to the rural community ATA (Additional file 3: Table S3). While previous pregnancy showed a lower trend of HPV when compared to those without previous pregnancy (Additional file 3: Table S3).

Compared with those who had no primary education revealed an APR of 1.25 (95% *CI*: 0.66–2.36) while secondary education appeared as an APR of 1.41 (95% *CI*: 0.70–2.86) (Additional file 3: Table S3). Farmers compared to non-farmers showed an increased APR of 1.55 (95% *CI*: 0.99–2.44) (Additional file 3: Table S3).

Alcohol consumption showed no association when compared to no consumption of alcohol, and a higher prevalence when smoking when referred to no smoking.

Farmer women were more likely to be positive for FGS and HPV infection compared to non-farmer women of the sample. Older age groups, tended to have low associations with FGS-HPV co-infection, and an increased number of gynaecological symptoms also revealed to be non-specific for FGS-HPV co-infection, with more than three symptoms showing lower APR.

## Discussion

This cross-sectional study reports a high prevalence of FGS, HPV, and the concomitant presence of the two conditions in women living in the Boeny region of north-western Madagascar. To the best of our knowledge, this is the first study describing the two conditions in the country together, bringing the attention to a relevant women’s health issue in Madagascar. An overall prevalence of 62.0% FGS and 43.0% HPV infection is reported in a primarily rural setting in a population that has never been vaccinated against HPV, mostly aligned with the prevalence estimated for the conditions in SSA [6, 59–61]. The concomitant presence of the two condition is 27.0%, as expected from the frequencies of the single infections.

Firstly, this data shows the urgency of implementing strategies for FGS screening, detection, and management in endemic areas. The policies and guidelines for the control of schistosomiasis mostly based on MDA [62] are systematically excluding the chronic forms of the disease. This does not only represent an obstacle for achieving universal health coverage [63] among the affected population, but also to the propagation of the disease hampering the WHO objective of eliminating schistosomiasis as a public health problem by 2030 [62]. At the current state of the art, biomedical and epidemiological factors associated with FGS remain unclear [10], as is the risk of developing the disease following *S. haematobium* infection [6, 10]. Given the high prevalence of FGS in the study area it is clear that more studies unravelling the natural history of the disease are needed, including more epidemiological studies to understand the burden of the disease in the entire country so as in other endemic countries.

Secondly, our results also show that HPV infection is highly prevalent in the population. Even if the majority of women (57.0%) is positive for the viral infection in the age range 18 to 24 years, still in higher age groups high positivity rates can be observed, suggesting a high risk for the subsequent development of CC or genital warts [7]. It is reported that on average, 90% of HPV infections can often be naturally cleared within a few years from acquisition [64], while the likelihood of newly acquired infections decreases with age [64]. In our study population, we observe a high proportion of women aged 35 years and older who tested positive, suggesting that specific factors (i.e., immunological, or other infections) may play a role in the clearance of infection or may be due to the reactivation of the infection. While the association of FGS with HIV has been previously described [17, 18], less is known about the association of FGS with HPV.

Our study shows a high concomitant presence of the two conditions (26.5%) but no evidence of association. Additional studies would be beneficial to confirm or further explore the role of FGS in HPV infection clearance but also the role of HPV in FGS progression and cure. Interestingly, in our study population, none of the women interviewed were vaccinated against HPV (Table 1). In Madagascar, just one project, between 2013 and 2015 [65], has been implemented to pilot the introduction of the HPV vaccination in the country in he rural district of Soaviandriana and in the urban district of Toamasina I. Following this project, no additional initiatives were promoted in the country for HPV vaccination and scattered initiatives are available for the overall CC prevention in the country. Worldwide it has been shown not only the impact of HPV vaccination in limiting the onset of CC [28, 66] but also the economic benefit for the countries and the individuals gained through HPV vaccination [67, 68]. Hence, our HPV prevalence data show clearly how urgently Madagascar needs to align with the WHO recommendations in terms of HPV and CC prevention [69]. In order to reach the 90-70-90 targets by 2030 [70], all countries need to adapt their strategies because, as the COVID-19 pandemic has shown, viruses easily cross borders hence “*nobody is safe until everyone is safe*”.

In our study, the majority of the population affected by FGS and HPV lives in rural areas. If for FGS this can be easily explained through the lack of access to clean water [71] for HPV this data is not common [72]. Nevertheless, it is noteworthy that global data on HPV infection are often based on urban populations, hence it remains unclear whether certain risk factors can be transferred from urban to rural contexts [72]. We can speculate that in our study, rural populations are more likely to engage in risky behaviours, as also suggested by Schluterman et al. [72], but it is also possible that a lack of prevention and general awareness of the disease plays a critical role. More studies are required to explore these elements in order to adapt awareness and prevention campaigns. In general, the lack of accessibility to health care services in rural communities [73] makes these data even more alarming because the management of the consequences of long-term HPV infections (namely, CC) can be more complicated in rural than in urban communities [74].

Notably, there is a lower association of HPV infection with previous pregnancy (Additional file 2: Table S2). We can speculate that as HPV is one of the most common sexually transmitted infections [75], in the specific context of our study, pregnancies can be associated with a lower number of sexual partners, which decreases the likelihood of infection [64]. Also, additional investigations in this direction might be useful to inform prevention and awareness strategies.

The diagnosis of FGS remains a challenge [76] especially because the implementation of colposcopy at primary level of care is complex [24]. In an attempt to build a screening algorithm to support the medical decision-making process, we associated signs and symptoms with the disease. Unfortunately, our data (Additional file 1: Table S1 and Additional file 2: Table S2) do neither show an association of FGS with specific symptoms, nor with a combination of symptoms. It is important to notice that the sample size limited the possibility to perform any probabilistic tests and our data remain purely descriptive. Although the power of the study is low to detect small differences, it would have been high enough to detect relevant differences suggesting that the establishment of a diagnostic algorithm based on clinical signs might be hard to establish. Nevertheless, given the importance of the outcome, it might be relevant to further investigate associations with an increased sample size even if the preliminary observations reported in the present study are not particularly promising.

Importantly, our study shows a very good acceptability of colposcopy at primary level of care (Fig. 1) indicating that besides the operational limitations, the availability of the service close to the patients in need is an approach that can be successfully adopted in the future. The dilemma of the implementation of advanced services in limited resources settings is delaying the possibility of offering appropriate care for all [63]. Our study shows the potential for the implementation of more specialised medical services in rural areas in a context with limited resources both in terms of infrastructures and trained staff. This calls for operational studies assessing feasibility and impact of such interventions in order to guarantee good health services for all.

It is noteworthy that our data show a high rate of miscarriages in the area (Tab.1) when compared to other SSA countries [77, 78]. Reproductive health is a key component of women’s health [79]. If not properly cared for, miscarriages can lead to serious socio-psychological [80] and medical sequalae [80]. In Madagascar, the health sector is particularly affected by the economic and political instability of the country [81, 82]. Interestingly, women’s health and specifically reproductive health is covered by the public health programs of the country [83] with increasing trends of improvement [84, 85]. Our data show alarming numbers, indicating that an overall reinforcement of women’s health services at primary level of care would not only benefit neglected conditions, such as FGS, HPV and CC, but also reproductive health more broadly. In addition, our data suggests the need for the country to reinforce surveillance on non-acute infections such as HIV. So far, in Madagascar the prevalence of HIV is estimated to be low [86] but, given that the epidemiological surveillance is not yet satisfactory [86], figures might differ from the real situation in the country. This could stimulate the conceptualization of more comprehensive integrated approaches addressing high burden infectious diseases, so far neglected in the country.

This study manages to highlight critical aspects of women’s health in Madagascar focusing on two conditions mostly neglected in the country but urgent on the global health agenda. Despite its strengths, this study does not come without limitations. A first limitation is given by the age of our study population (aged 18–49), which does not allow to sufficiently explore the effects of FGS and HPV on CC cancer onset and prevalence, since it normally occurs at older ages [70]. Additionally, the limited sample size partly prevented more advanced statistical analysis. The lack of identifying any association of the studied conditions with any of the risk factors analysed strongly limits the possibility to provide recommendations for preventive and control strategies in Madagascar so as in other similar contexts.

## Conclusions

Our study highlights the high prevalence of FGS and HPV infections and important gaps in their management among women in Madagascar. There is an urgent need to establish services that can align Madagascar’s public health agenda with that of the global health community. Strengthening primary health care services to address FGS and HPV can improve overall women's health in Madagascar, and in other contexts with similar epidemiological profiles and resource availability.

### Supplementary Information

**Additional file 1: Table S1.** Regression analysis FGS.

**Additional file 2: Table S2.** Regression analysis HPV.

**Additional file 3: Table S3.** Regression analysis FGS and HPV.

## Data Availability

Additional datasets used during the current study are available from the corresponding author on reasonable request and will be freely available to researchers who wish to use them for non-commercial purposes, without breaching the confidentiality of participants. All relevant data is already visible in the manuscript.

## Abbreviations

APR: Adjusted prevalence ratio
CPR: Crude prevalence ratio
CC: Cervical cancer
COLIM: Colposcopic image
*CI*: Confidence interval
CRF: Case report form
CVL: Cervical vaginal lavage
DNA: Deoxyribonucleic acid
FGS: Female genital schistosomiasis
HPV: Human papilloma virus
HR-HPV: High risk human papilloma virus (infection)
LMIC: Low- and middle-income country
MDA: Mass drug administration
NCD: Non communicable disease
NTD: Neglected tropical disease
PCR: Polymerase chain reaction
PHCC: Primary health care center
PR: Prevalence ratio
PZQ: Praziquantel
SDG: Sustainable development goal
SSA: Sub Saharan Africa
WASH: Water, sanitation, and hygiene
WHO: Word Health Organization
UN: United Nations
