# Supplementary material for: Female genital schistosomiasis, human papilloma virus infection, and cervical cancer in rural Madagascar: a cross sectional study
Source: Infect Dis Poverty. 2023 Sep 25;12:89. doi: 10.1186/s40249-023-01139-3 (PMC10518971; doi:10.1186/s40249-023-01139-3)
Supplement: Supplementary file 1 — Additional file 1: Table S1. Regression analysis FGS. [file 40249_2023_1139_MOESM1_ESM.docx]

| **Additional file 1:** Regression analysis for FGS | | | | | |  |
| --- | --- | --- | --- | --- | --- | --- |
| **Characteristic** | *overall participants* | *FGS positive participants (n)* | *FGS positivity among participants (%)* | *CPR*  *(95% CI)* | *APR*  *(95% CI)* |  |
|  |  |  |  |  |  |  |
| **HPV** |  |  |  |  |  |  |
| negative | 173 | 109 | 63.0 | **Ref** | **Ref** |  |
| positive | 129 | 80 | 62.0 | 1.0 (0.83; 1.2) | 1.0 (0.82; 1.2) |  |
| **Age** |  |  |  |  |  |  |
| 18-24 | 75 | 47 | 63.0 | **Ref** | **Ref** |  |
| 25-34 | 113 | 66 | 58.0 | 0.9 (0.73; 1.2) | 0.9 (0.70; 1.1) |  |
| 35-44 | 73 | 50 | 69.0 | 1.1 (0.86; 1.4) | 1.0 (0.83; 1.3) |  |
| 45+ | 41 | 26 | 63.0 | 1.0 (0.75; 1.3) | 0.9 (0.68; 1.2) |  |
| **PHCC** |  |  |  |  |  |  |
| Antanambao Andranolava | 111 | 76 | 69.0 | **Ref** | **Ref** |  |
| Ankazomborona | 50 | 40 | 80.0 | 1.2 (1.0; 1.4) | 1.2 (1.0; 1.6) |  |
| Marovoay | 141 | 73 | 52.0 | 0.8 (0.6; 0.9) | 0.8 (0.6; 1.1) |  |
| **Previous Treatment** |  |  |  |  |  |  |
| No | 200 | 120 | 60.0 | **Ref** | **Ref** |  |
| Yes | 97 | 67 | 69.0 | 1.2 (1.0; 1.4) | 1.0 (0.8; 1.2) |  |
| Do not know | 5 | 2 | 40.0 | 0.7 (0.2; 2.0) | 0.6 (0.2; 1.6) |  |
| **Education** |  |  |  |  |  |  |
| No education | 34 | 21 | 62.0 | **Ref** | **Ref** |  |
| Primary education | 145 | 101 | 70.0 | 1.1 (0.9; 1.5) | 1.1 (0.8; 1.5) |  |
| Secondary education and higher | 123 | 67 | 55.0 | 0.9 (0.7; 1.2) | 1.0 (0.7; 1.4) |  |
| **Profession** |  |  |  |  |  |  |
| Non- Farmer | 129 | 72 | 55.0 | **Ref** | **Ref** |  |
| Farmer | 173 | 117 | 68.0 | 1.2 (1.0; 1.5) | 1.0 (0.8; 1.2) |  |
| **Previous pregnancy** |  |  |  |  |  |  |
| No pregnancy | 26 | 11 | 42.0 | **Ref** | **Ref** |  |
| Previous pregnancy | 276 | 178 | 65.0 | 1.5 (1.0; 2.4) | 1.4 (0.9; 2.3) |  |
| **Number of symptoms** |  |  |  |  |  |  |
| No symptoms | 100 | 71 | 71.0 | **Ref** | **Ref** |  |
| 1 symptom | 97 | 57 | 59.0 | 0.8 (0.7; 1.0) | 0.9 (0.7; 1.1) |  |
| 2 symptoms | 54 | 34 | 63.0 | 0.9 (0.7; 1.1) | 1.0 (0.8; 1.3) |  |
| 3 and more symptoms | 51 | 27 | 51.0 | 0.8 (0.6; 1.0) | 0.8 (0.6; 1.0) |  |

***Abbreviations*:** *APR: adjusted prevalence ratio*; *CPR: crude prevalence ratio; CI: Confidence interval; CL: Confidence limit; HPV: Human Papilloma Virus; FGS: Female Genital Schistosomiasis; PHCC*: *Primary Healthcare Centre;* *PR: prevalence ratio; Ref: Reference category*
