# Supplementary material for: Female genital schistosomiasis, human papilloma virus infection, and cervical cancer in rural Madagascar: a cross sectional study
Source: Infect Dis Poverty. 2023 Sep 25;12:89. doi: 10.1186/s40249-023-01139-3 (PMC10518971; doi:10.1186/s40249-023-01139-3)
Supplement: Supplementary file 2 — Additional file 2: Table S2. Regression analysis HPV. [file 40249_2023_1139_MOESM2_ESM.docx]

| **Additional file 2:** Regression analysis for HPV | | | | | |  |
| --- | --- | --- | --- | --- | --- | --- |
| **Characteristic** | *overall participants* | *HPV positive participants (n)* | *HPV positivity among participants (%)* | *CPR*  *(95% CI)* | *APR*  *(95% CI)* |  |
|  |  |  |  |  |  |  |
| **FGS** |  |  |  |  |  |  |
| negative | 113 | 49 | 43.0 | **Ref** | **Ref** |  |
| positive | 189 | 80 | 42.0 | 1.0 (0.8; 1.3) | 1.0 (0.7; 1.3) |  |
| **Age** |  |  |  |  |  |  |
| 18-24 | 75 | 43 | 57.0 | **Ref** | **Ref** |  |
| 25-34 | 113 | 38 | 34.0 | 0.6 (0.4; 0.8) | 0.6 (0.45; 0.89) |  |
| 35-44 | 73 | 33 | 45.0 | 0.8 (0.6; 1.1) | 0.9 (0.6; 1.2) |  |
| 45+ | 41 | 15 | 37.0 | 0.6 (0.4; 1.0) | 0.7 (0.4; 1.1) |  |
| **PHCC** |  |  |  |  |  |  |
| Antanambao Andranolava | 111 | 49 | 44.0 | **Ref** | **Ref** |  |
| Ankazomborona | 50 | 23 | 46.0 | 1.0 (0.7; 1.5) | 1.1 (0.7; 1.6) |  |
| Marovoay | 141 | 57 | 40.0 | 0.9 (0.7; 1.2) | 0.9 (0.6; 1.3) |  |
| **Education** |  |  |  |  |  |  |
| No education | 34 | 13 | 38,00 | **Ref** | **Ref** |  |
| Primary education | 145 | 59 | 41,00 | 1.1 (0.7; 1.7) | 1.1 (0.7; 1.8) |  |
| Secondary education and higher | 123 | 57 | 46,00 | 1.2 (0.8; 1.9) | 1.3 (0.8; 2.2) |  |
| **Profession** |  |  |  |  |  |  |
| Non- Farmer | 129 | 45 | 44.0 | **Ref** | **Ref** |  |
| Farmer | 173 | 75 | 43.0 | 1.0 (0.8; 1.4) | 1.1 (0.8; 1.6) |  |
| **Previous pregnancy** |  |  |  |  |  |  |
| No pregnancy | 26 | 18 | 69.0 | **Ref** | **Ref** |  |
| Previous pregnancy | 276 | 111 | 40.0 | 0.6 (0.4; 0.8) | 0.7 (0.5; 0.9) |  |
| **Smoking** |  |  |  |  |  |  |
| No smoking | 282 | 119 | 42.0 | **Ref** | **Ref** |  |
| smoking | 20 | 10 | 50.0 | 1.2 (0.8; 1.9) | 1.2 (0.8; 2.0) |  |
| **Number of symptoms** |  |  |  |  |  |  |
| No symptoms | 100 | 44 | 44.0 | **Ref** | **Ref** |  |
| 1 symptom | 97 | 45 | 46.0 | 1.1 (0.8; 1.4) | 1.0 (0.8; 1.4) |  |
| 2 Symptoms | 54 | 22 | 41.0 | 0.9 (0.6; 1.4) | 0.9 (0.6; 1.4) |  |
| 3 and more symptoms | 51 | 18 | 35.0 | 0.8 (0.5; 1.2) | 0.8 (0.5; 1.2) |  |
| **Alcohol** |  |  |  |  |  |  |
| No alcohol consumption | 217 | 92 | 42.0 | **Ref** | **Ref** |  |
| Alcohol consumption | 85 | 37 | 44.0 | 1.0 (0.8; 1.4) | 1.1 (0.8; 1.5) |  |

***Abbreviations*:** *APR: adjusted prevalence ratio*; *CPR: crude prevalence ratio; CI: Confidence interval; CL: Confidence limit; HPV: Human Papilloma Virus; FGS: Female Genital Schistosomiasis; PHCC*: *Primary Healthcare Centre;* *PR: prevalence ratio; Ref: Reference category*
