# Supplementary material for: Female genital schistosomiasis, human papilloma virus infection, and cervical cancer in rural Madagascar: a cross sectional study
Source: Infect Dis Poverty. 2023 Sep 25;12:89. doi: 10.1186/s40249-023-01139-3 (PMC10518971; doi:10.1186/s40249-023-01139-3)
Supplement: Supplementary file 3 — Additional file 3: Table S3. Regression analysis FGS and HPV. [file 40249_2023_1139_MOESM3_ESM.docx]

| **Additional file 3:** Regression analysis for FGS and HPV co-presence | | | | | |  |
| --- | --- | --- | --- | --- | --- | --- |
| **Characteristic** | *overall participants* | *HPV positive participants (n)* | *HPV positivity among participants (%)* | *CPR*  *(95% CI)* | *APR*  *(95% CI)* |  |
|  |  |  |  |  |  |  |
| **Age** |  |  |  |  |  |  |
| 18-24 | 75 | 26 | 35.0 | **Ref** | **Ref** |  |
| 25-34 | 113 | 21 | 19.0 | 0.5 (0.3; 0.9) | 0.6 (0.33; 0.89) |  |
| 35-44 | 73 | 23 | 32.0 | 0.9 (0.6; 1.4) | 1.0 (0.6; 1.6) |  |
| 45+ | 41 | 10 | 24.0 | 0.7 (0.4; 1.3) | 0.6 (0.3; 1.2) |  |
| **PHCC** |  |  |  |  |  |  |
| Antanambao Andranolava | 111 | 34 | 31.0 | **Ref** | **Ref** |  |
| Ankazomborona | 50 | 18 | 36.0 | 1.2 (0.7; 1.9) | 1.3 (0.8; 2.2) |  |
| Marovoay | 141 | 28 | 20.0 | 0.7 (0.4; 1.0) | 0.7 (0.4; 1.3) |  |
| **Education** |  |  |  |  |  |  |
| No education | 34 | 8 | 24.0 | **Ref** | **Ref** |  |
| Primary education | 145 | 41 | 28.0 | 1.2 (0.6; 2.3) | 1.3 (0.7; 2.4) |  |
| Secondary education and higher | 123 | 31 | 25.0 | 1.1 (0.5; 2.1) | 1.41 (0.7; 2.9) |  |
| **Profession** |  |  |  |  |  |  |
| Non- Farmer | 129 | 21 | 20.0 | **Ref** | **Ref** |  |
| Farmer | 173 | 54 | 31.0 | 1.6 (1.0; 2.3) | 1.6 (1.0; 2.4) |  |
| **Previous pregnancy** |  |  |  |  |  |  |
| No pregnancy | 26 | 8 | 31.0 | **Ref** | **Ref** |  |
| Previous pregnancy | 276 | 72 | 26.0 | 0.9 (0.5; 1.6) | 0.8 (0.4; 1.7) |  |
| **Smoking** |  |  |  |  |  |  |
| No smoking | 282 | 74 | 26.0 | **Ref** | **Ref** |  |
| Smoking | 20 | 6 | 30.0 | 1.1 (0.6; 2.3) | 1.3 (0.6; 2,7) |  |
| **Number of Symptoms** |  |  |  |  |  |  |
| None | 100 | 29 | 29.0 | **Ref** | **Ref** |  |
| 1 symptom | 97 | 28 | 29.0 | 1.0 (0.6; 1.5) | 1.0 (0.7; 1.6) |  |
| 2 symptoms | 54 | 15 | 28.0 | 1.0 (0.6; 1.6) | 1.1 (0.6; 1.8) |  |
| 3 and more symptoms | 51 | 8 | 16.0 | 0.5 (0.3; 1.1) | 0.5 (0.2; 1.0) |  |
| **Alcohol** |  |  |  |  |  |  |
| No alcohol consumption | 217 | 59 | 27.0 | **Ref** | **Ref** |  |
| Alcohol consumption | 85 | 21 | 25.0 | 0.9 (0.6; 1.4) | 1.0 (0.7; 1.6) |  |

***Abbreviations*:** *APR: adjusted prevalence ratio*; *CPR: crude prevalence ratio; CI: Confidence interval; CL: Confidence limit; HPV: Human Papilloma Virus; FGS: Female Genital Schistosomiasis; PHCC*: *Primary Healthcare Centre;* *PR: prevalence ratio; Ref: Reference category*
